# Neuro-symbolic representation learning on biological knowledge graphs

**DOI:** 10.1093/bioinformatics/btx275

**Published:** 2017-04-25

**Authors:** Mona Alshahrani, Mohammad Asif Khan, Omar Maddouri, Akira R Kinjo, Núria Queralt-Rosinach, Robert Hoehndorf

**Affiliations:** 1 Computer, Electrical and Mathematical Sciences & Engineering Division, Computational Bioscience Research Center, King Abdullah University of Science and Technology, Thuwal, Kingdom of Saudi Arabia; 2 Life Sciences Division, College of Science & Engineering, Hamad Bin Khalifa University, HBKU, Doha, Qatar; 3 Institute for Protein Research, Osaka University 3-2 Yamadaoka, Suita, Osaka, Japan; 4 Department of Integrative Structural and Computational Biology, The Scripps Research Institute, La Jolla, CA, USA

## Abstract

**Motivation:**

Biological data and knowledge bases increasingly rely on Semantic Web technologies and the use of knowledge graphs for data integration, retrieval and federated queries. In the past years, feature learning methods that are applicable to graph-structured data are becoming available, but have not yet widely been applied and evaluated on structured biological knowledge. Results: We develop a novel method for feature learning on biological knowledge graphs. Our method combines symbolic methods, in particular knowledge representation using symbolic logic and automated reasoning, with neural networks to generate embeddings of nodes that encode for related information within knowledge graphs. Through the use of symbolic logic, these embeddings contain both explicit and implicit information. We apply these embeddings to the prediction of edges in the knowledge graph representing problems of function prediction, finding candidate genes of diseases, protein-protein interactions, or drug target relations, and demonstrate performance that matches and sometimes outperforms traditional approaches based on manually crafted features. Our method can be applied to any biological knowledge graph, and will thereby open up the increasing amount of Semantic Web based knowledge bases in biology to use in machine learning and data analytics.

**Availability and implementation:**

https://github.com/bio-ontology-research-group/walking-rdf-and-owl

**Supplementary information:**

Supplementary data are available at *Bioinformatics* online.

## 1 Introduction

The Semantic Web (Berners-Lee *et al.*, 2001), a project with the stated purpose of forming a consistent logical and meaningful web of data using semantic technologies to make data machine-understandable and processable, has been highly successful in biology and biomedicine (Katayama *et al.*, 2014). Many major bioinformatics databases now make their data available as Linked Data in which both biological entities and connections between them are identified through a unique identifier (an Internationalized Resource Identifier or IRI) and the connections between them are expressed through standardized relations (Smith *et al.*, 2005; Wood *et al.*, 2014). Linked Data can enable interoperability between multiple databases simply by reusing identifiers and utilizing no-SQL query languages such as SPARQL (Seaborne and Prud’hommeaux, 2008) that can perform distributed queries over multiple databases. Some of the first major efforts to make life science data available as Linked Data have been the UniProt RDF initiative (The UniProt Consortium, 2015) and the Bio2RDF project (Belleau *et al.*, 2008; Callahan *et al.*, 2013). UniProt focuses on making data within a single database, UniProt, available as Linked Data so that information and identifiers can be reused in other databases, while Bio2RDF has the aim to combine multiple databases and demonstrate the potential of Linked Data in life sciences, in particular with regard to provenance tracking, usability and interoperability. Now, major databases, such as those provided by the European Bioinformatics Institute (EBI) and the National Center for Biotechnology Information (NCBI), are made available as Linked Data (Jupp *et al.*, 2014; Kim *et al.*, 2016). Additionally, community guidelines and principles for data publishing such as the FAIR principles (Wilkinson *et al.*, 2016) require data to be made available in a way that is amenable to interoperability through linking and federation of queries.

A second major component of applications of the Semantic Web in the life sciences has been the development and use of ontologies. Ontologies are specifications of a conceptualization of a domain (Gruber, 1995), i.e. they formally and explicitly specify some of the main regularities (classes of entities) that can be found within a domain and their interconnections (Hoehndorf *et al.*, 2015b). Ontologies are now widely used in biological datasets for the annotation and provision of metadata. They are commonly represented in formal languages with model theoretic semantics (Grau *et al.*, 2008; Horrocks, 2007) which makes them amenable to automated reasoning. However, the large size of the ontologies and the complexity of the languages and reasoning tasks involved have somewhat limited the use of ontologies in automated reasoning. In particular, there is still a large disparity between the ontologies in biomedicine and the databases that uses them for annotation in the sense that they are rarely integrated within the same data model. While inferences over the ontologies, as part of ontology development and quality assurance process, become increasingly common (Mungall *et al.*, 2012; The Gene Ontology Consortium, 2015), they are not always applied to infer new relations between biomedical data.

Recently, several machine learning methods have become available that can be utilized to learn features from raw data (Lecun *et al.*, 2015). Several of these methods can also be applied to graph-structured data (Perozzi *et al.*, 2014; Yanardag and Vishwanathan, 2015). While most of these methods are developed for graphs without edge labels (in contrast to Linked Data in which edge labels represent the type of relation between entities), some methods have also been extended to incorporate edge labels (Ristoski and Paulheim, 2016). However, to be applicable to biological data, a crucial aspect is the interoperability between both the data layer (as expressed in Linked Data formats) and the annotations of data items or semantic layer (expressed through ontologies and the background knowledge they provide). This tight integration between data and knowledge, as dominantly present in biological databases, benefits from automated reasoning so that it becomes possible to consider inferred knowledge, handle data consistency and identify incompatible conceptualizations.

We developed a method to leverage the semantic layer in knowledge graphs such as the Semantic Web or Wikidata by combining automated reasoning over ontologies and feature learning with neural networks, to generate vector representations of nodes in these graphs (node embeddings). We demonstrate that these representations can be used to predict edges with biological meaning. In particular, we demonstrate that our approach can predict disease genes, drug targets, drug indications, gene functions and other associations with high accuracy, in many cases matching or outperforming state of the art methods.

Our results demonstrate how Linked Data and ontologies can be used to form biological knowledge graphs in which heterogeneous biological data and knowledge are combined within a formal framework, and that these graphs can not only be used for data retrieval and search, but provide a powerful means for data analysis and discovery of novel biological knowledge.

## 2 Materials and methods

### 2.1 Data description

In our experiments, we build a knowledge graph based on three ontologies: the Gene Ontology (GO) (Ashburner *et al.*, 2000) downloaded on 18 July 2016, the Human Phenotype Ontology (Köhler *et al.*, 2014) downloaded on 18 July 2016, and the Disease Ontology (Kibbe *et al.*, 2014) downloaded on 19 August 2016. We also use the following biological databases in our knowledge graph:

- Human GO annotations from SwissProt (The UniProt Consortium, 2015), and phenotype annotations from the HPO databases (Köhler *et al.*, 2014), downloaded on 23 July 2016. We include a total of 212 078 GO annotations and 153 575 phenotype annotations.
- Human Proteins interactions from the STRING database (Szklarczyk *et al.*, 2011) downloaded on 18 July 2016. We filter proteins by their interactions confidence score and choose those above 700. The total number of interactions in this dataset is 188 424.
- Human chemical–protein interactions downloaded from the STITCH database (Kuhn *et al.*, 2012), on 28 August 2016, filtered for confidence score of 700. The total number of drug-target interactions present in the graph is 335 780.
- Genes and disease associations from DisGeNET (Piñero *et al.*, 2015), downloaded on 28 August 2016, consisting of 236 259 associations.
- Drug side effects and indications from SIDER (Kuhn *et al.*, 2010), downloaded on 15 August 2016. We include a total of 54 806 drug–side effect pairs and 6159 drug–indication pairs in our graph.
- Diseases and their phenotypes from the HPO database (Köhler *et al.*, 2014) and text mining (Hoehndorf *et al.*, 2015a). We include a total of 84 508 phenotype annotations of diseases.

We map all protein identifiers to Entrez gene identifiers and use these to represent both genes and proteins. We use PubChem identifiers to represent chemicals and we map UMLS identifiers associated with diseases in DisGeNET and indications in SIDER to the Disease Ontology using mappings provided by Disease Ontology. We further map UMLS identifiers associated with side effects in SIDER to HPO identifiers using mapping between UMLS and HPO (Hoehndorf *et al.*, 2014).

A knowledge graph is a graph-based representation of entities in the world and their interrelations. Knowledge graphs are widely used to facilitate and improve search, and they are increasingly being developed and used through Semantic Web technologies such as the Resource Description Framework (RDF) (Candan *et al.*, 2001). Here, we focus on knowledge graphs centered around biological entities and their interactions, ignoring all meta-data including labels or provenance. The knowledge graphs we consider have two distinct types of entities: biological entities, and classes from biomedical ontologies that provide background knowledge about a domain. The aim of building a biological knowledge graph is to represent, within a single formal structure, biological relations between entities, their annotations with biological ontologies, and the background knowledge in ontologies.

We make a clear distinction between instances and classes. While there is some debate about which kinds of biological entities should be treated as instances and which as classes (Smith *et al.*, 2005), similarly to other Linked Data approaches (The UniProt Consortium, 2015), we treat biological entities such as types of proteins, diseases, or chemicals, as instances in the knowledge graph. In our case, classes from the Disease Ontology are also treated as instances. On the level of instances, we can integrate existing graph-based representations used in biology and biomedicine, in particular biological networks such as protein-protein interaction networks, genetic interaction networks, metabolic interactions or pathways.

Ontology-based annotations are expressed by asserting a relation between the instance (e.g. a disease or protein) and an instance of the ontology class. For example, we express the information that the protein *Foxp2* has the function *transcription factor binding* (GO:0003700) by the two axioms $hasFunction(foxp2,f_{1})$ and $instanceOf(f_{1},GO:0003700)$ where *foxp*2 and *f*_1_ are instances, GO:0003700 the class http://purl.obolibrary.org/obo/GO_0003700 in GO, *hasFunction* an object property, and *instanceOf* the rdf:type property specified in the OWL standard (OWL Working Group, 2009) as expressing an instantiation relation. The instance *f*_1_ can be expressed as an anonymous instance (i.e. a blank node in the RDF representation) or be assigned a unique new IRI. In our knowledge graph, we create a new IRI (i.e. an IRI that does not occur anywhere else in the graph) for each of these instances.

### 2.2 Ontology-based classification

Due to the large size of the knowledge graphs we process, we rely on polynomial-time automated reasoning methods. OWL provides three profiles (Motik *et al.*, 2009) that facilitate polynomial time inferences, and multiple RDF stores implement different subsets of OWL to facilitate inferences and improve querying. For example, the OWL-Horst subset (ter Horst, 2005) is used by several RDF stores and is useful in data management and querying. In biological and biomedical ontologies, the OWL 2 EL profile is widely used to develop the large ontologies that are in use in the domain, and has been found to be useful and sufficient for a large number of tasks (Hoehndorf *et al.*, 2011; Mungall *et al.*, 2012; Suntisrivaraporn *et al.*, 2007).

OWL 2 EL supports basic inferences over ontologies’ class hierarchies (including intersection, existential quantification and disjointness between named classes), supports inferences over object properties (transitivity, reflexivity and object property composition) and can infer the classification of instances. We make use of OWL 2 EL for representing the knowledge graphs we generate and utilize the ELK reasoner (Kazakov *et al.*, 2014) for automated reasoning over them. In principle, other profiles of OWL can also be used following a similar approach, but may not be feasible due to the high computational complexity of generating inferences (Baader *et al.*, 2003). OWL 2 EL supports the following class descriptions, class and object property axioms (using capital letters for classes, lower case letters for object properties, and $x_{1},x_{2},\ldots$ for instances):

- Class description: class intersection (A⊓B), existential quantification (∃r.A), limited enumeration using a single instance ($\left\{x_{1}\right\}$)
- Class axioms: subclass (A⊑B), equivalent class (A≡B), disjointness (A⊓B⊑⊥)
- Object property axioms: sub-property (r⊑s), property chains (r ○ s⊑q), equivalent property (r≡s), transitive properties (r ○ r⊑r), reflexive properties

We deductively close the knowledge graph with respect to the OWL 2 EL profile, using an OWL 2 EL reasoner (Kazakov *et al.*, 2014). A knowledge graph KG is deductively closed if and only if for all ϕ such that KG⊨ϕ, ϕ∈KG. In general, the deductive closure of a knowledge is countably infinite. Therefore, we only add inferences that can be represented explicitly as edges between named individuals and classes in KG, i.e. between entities that are explicitly named in KG. In particular, for all instances $x_{i},x_{j}\in KG$ and object properties r∈KG, if $KG⊨r(x_{i},x_{j})$, then $r(x_{i},x_{j})\in KG^{⊨}$. Furthermore, for all named classes C∈KG and instances x∈KG, if KG⊨C(x), then $C(x)\in KG^{⊨}$. Finally, we also infer relations between classes, in particular subclass axioms, and add them to the inferred graph: for any class C,D∈KG, if KG⊨C⊑D, then $C⊑D\in KG^{⊨}$.

We use the OWL API version 4 (Horridge *et al.*, 2007) to classify the input knowledge graph and add all inferences obtained by using the ELK reasoner as new edges to the knowledge graph to generate $KG^{⊨}$. We use this fully inferred graph as a basis for generating the node embeddings through our method.

### 2.3 Walking RDF and OWL

To generate node embeddings, we use a modified version of the DeepWalk algorithm (Perozzi *et al.*, 2014) in which we consider edge labels as part of the walk. A random walk of length *n* over a graph G=(V,E) and start vertex $v_{0}\in V$ is an ordered sequence of vertices $(v_{0},\ldots ,v_{n}),\;v_{i}\in V$, and each *v_i_* (*i* > 0) is determined by randomly selecting an adjacent node of $v_{i-1}$. As knowledge graphs generated by our method additionally have edges of different types (i.e. edge labels, ℓ(E)), we extend this notion to edge-labeled random walks. An edge-labeled random walk of length *n* over the graph G=(V,E), edge labels ℓ:E↦L in the label space *L* (i.e. the set of object properties in the knowledge graph underlying *G*), and start vertex $v_{0}\in V$ is a sequence $\left(v_{0},l_{1},v_{1},\ldots ,l_{n},v_{n}\right)$ such that $v_{i}\in V,\;l_{i}\in L$, and, starting with *v*_0_ and for all *v_i_* (*i *<* n*), a random outgoing edge $e_{i+1}$ of *v_i_*, ending in $v_{i+1}$ is chosen to generate $l_{i+1}$ from $\ell (e_{i+1})$ and $v_{i+1}$.

We implement this algorithm as an extension of the DeepWalk (Perozzi *et al.*, 2014). The algorithm takes a knowledge graph G=(V,E) as input and generates a corpus C consisting of a set of edge-labeled random walks, starting either from all vertices v∈V, or all vertices v∈U of a specified subset of U⊆V. Parameters of the algorithm are the length of the walks and the number of walks per node. Source code of the algorithm and documentation are freely available at https://github.com/bio-ontology-research-group/walking-rdf-and-owl.

### 2.4 Learning embeddings

We use the corpus C of edge-labeled random walks as an input for learning embeddings of each node. We follow the skip-gram model (Mikolov *et al.*, 2013) to generate these embeddings. Given a sequence of words, $\left(w_{1},\ldots ,w_{N}\right)$ in C, a skip-gram model aims to maximize the average log probability

(1) $$\frac{1}{N}\sum_{n=1}^{N}\sum_{-c\leq j\leq c,j\neq 0}\;\text{log}\;p(w_{n+j}|w_{n})$$

in which *c* represents a context or window size. To define $p(w_{n+j}|w_{n})$, we use negative sampling, following (Mikolov *et al.*, 2013), i.e. replacing $\text{log}\;p(w_{O}|w_{I})$ above with a function to discriminate target words (*w_O_*) from a noise distribution $P_{n}(w)$ (Mikolov *et al.*, 2013), drawing *k* words from $P_{n}(w)$:

(2) $$\log\sigma (v_{w_{O}}^{\prime ⊺}v_{w_{I}})+\sum_{i=1}^{k}E_{w_{i}\sim P_{n}(w)}\left[\log\sigma (-v_{w_{i}}^{\prime ⊺}v_{w_{I}})\right]$$

The vector representation (embedding) of a word *s* occurring in corpus *C* is the vector *v_s_* in Eq. 2 derived by maximizing Eq. 1. The dimension of this vector is a parameter of the method.

Since our corpus consists of often repeated edge labels (due to the relatively small size of the label space L), we further use sub-sampling of frequent words (Mikolov *et al.*, 2013) (which mainly represent edge labels in the corpora we generate) to improve the quality of node embeddings. We follow (Mikolov *et al.*, 2013) and discard, during training, each word *w_i_* (i.e. node or edge) with a probability $P(w_{i})=1-\sqrt{\frac{t}{f(w_{i})}}$ where *t* is a threshold parameter.

It is obvious from this formulation that the parameters for learning the representation of nodes in a knowledge graph include the number of walks to perform for each vertex, the length of each individual walk, a subset *U* of vertices from which to start walks, the size of the vector representations learned by the skip-gram model, the window or context size employed in the skip-gram model, the parameter *t* used to sub-sample frequent words (we use $t=10^{-3}$ for all our experiments), and the number of words to draw from the noise distribution (we fix this parameter to 5 in our experiments). There are several additional parameters for training a skip-gram model, including learning rate and certain processing steps on the corpus, for which we chose default values in the gensim (https://radimrehurek.com/gensim/) skip-gram implementation.

### 2.5 Prediction

The embeddings can be used as features in machine learning tasks that should encode for the local neighborhood of each node, thereby encoding for the (local) information contained in a knowledge graph about a certain vertex. We apply these features to the task of edge prediction, in which we aim to estimate the probability that an edge with label *l* exists between vertices *v*_1_ and *v*_2_ given their vector representation, $𝕧(v_{1})$ and $𝕧(v_{2})$: $p((v_{1},v_{2},l)\in E|\langle 𝕧(v_{1}),𝕧(v_{2})\rangle )$. We use the logistic regression classifier implemented in the sklearn library (Pedregosa *et al.*, 2011) to train logistic regression models.

We build separate binary prediction models for each object property in the knowledge graphs. For model building and testing, we employ 5-fold cross-validation. For each object property representing edge label *l*, cross-validation folds are built by randomly removing 20% of edges with label *l* in the knowledge graph, then applying deductive inference, corpus generation through edge-labeled random walks, learning of vector representations of nodes, and building of a binary logistic regression model. The degree distribution in our knowledge graph before and after removing 20% of edges is available as Supplementary Material. A model for edges with label *l* is trained using as positive instances all pairs of vertices for which an edge with label *l* exists in the modified knowledge graph (in which 20% of edges with label *l* have previously been removed), and using as negatives a random subset of all pairs of vertices $\left(v_{r_{1}},v_{r_{2}}\right)$ such that $v_{r_{1}}$ is of the same type (i.e. an instance of the same class in the knowledge graph) as all sources of edges with label *l*, and $v_{r_{2}}$ is of the same type as all targets of edges with label *l*. For example, if edges with label *l* are all between instances of *Drug* and *Disease* in a knowledge graph, then we sub-sample negative instances among all pairs of instances of *Drug* and *Disease* for which no edge exists in the original knowledge graph. The constraint of choosing negatives from the same general types of entities is necessary because instances of different types will be clearly separable within the embeddings, and evaluation using those would therefore bias the results. We randomly generate a set of negative samples with the same cardinality as the set of positive samples, both for model training and prediction. A limitation of our choice of negatives is that some edges we consider as negatives may not be true negatives due to the likely incompleteness of the knowledge graph and the sources we used for its generation.

The embeddings can also be used for findings similar nodes using a measure of similarity. We use cosine similarity to compute the similarity between two vectors: $sim(v_{1},v_{2})=\frac{v_{1}\cdot v_{2}}{\left||A||\;\;||B|\right|}$

### 2.6 Parameter optimization

Using the performance on the final prediction model, we perform parameter optimization through a limited grid search. We only optimize embedding size, number of walks, walk length and context size for the skip-gram model through a grid search since an exhaustive optimization would be too computationally expensive. Furthermore, we only use a single object property to test how results change with each choice of parameter, due to computational constraints. We tested the following 625 parameters: embedding sizes of 32, 64, 128, 256 and 512, number of walks 50, 100, 200, 300 and 500, walk length 5, 10, 15, 20 and 30, and skip-gram context sizes 5, 10, 15, 20 and 30. We found the best performing parameters to be 512 for the embedding size, 100 for the number of walks, 20 for the walks length and 10 for the skip-gram context size, and we fix these parameters throughout our experiments.

## 3 Results

### 3.1 Neuro-symbolic feature learning using Semantic Web technologies

We build a knowledge graph using Semantic Web technologies centered on human biomedical data. The graph incorporates several biological and biomedical datasets and is split in two layers, instances and classes. On the level of instances in the knowledge graph, we combine protein-protein interactions (PPIs) (Szklarczyk *et al.*, 2011), chemicals (drugs) and their protein targets (Kuhn *et al.*, 2012), drugs and their indications (Kuhn *et al.*, 2010), and genes and the diseases they are involved in (Piñero *et al.*, 2015). On the level of classes, we include the Human Phenotype Ontology (Köhler *et al.*, 2014), and the Gene Ontology (Ashburner *et al.*, 2000), and we include annotations of diseases and their phenotypes (Hoehndorf *et al.*, 2015a; Köhler *et al.*, 2014), genes and their phenotypes (Köhler *et al.*, 2014), and human protein functions and subcellular locations (The UniProt Consortium, 2015). The knowledge graph, including the data, ontologies and our formal representation of ontology-based annotations, consists of 7 855 737 triples. We use the Elk reasoner (Kazakov *et al.*, 2014) to deductively close this graph, and through the application of ontology-based inference, we further infer 5 616 273 new triples and add them to the knowledge graph.

We utilize this knowledge graph as the input to our algorithm that can learn representations of nodes. These representations represent the neighborhood of a node as well as the kind of relations that exist to the neighboring nodes. To learn these representations, we perform random walks from each node in the knowledge graph repeatedly, use the resulting walks as sentences within a corpus, and apply the Word2Vec skip-gram model (Mikolov *et al.*, 2013) to learn embeddings for each node.

We use the fully inferred, deductively closed knowledge graph to perform the random walks. Performing random walks on the deductively closed graph has the advantage that not only asserted axioms will be taken into consideration, but representations can also include inferred knowledge that is not present explicitly in the graph. For example, for an assertion that a gene *g* has a function *F* (where *F* is a class in the GO), all superclasses of *F* in GO will be added as annotations to *g*; sub-properties (such as binds⊆interacts-with) asserted in an ontology or database will be resolved; transitive, reflexive object properties and property chains resolved and the inferred edges added.

**Fig. 1:**
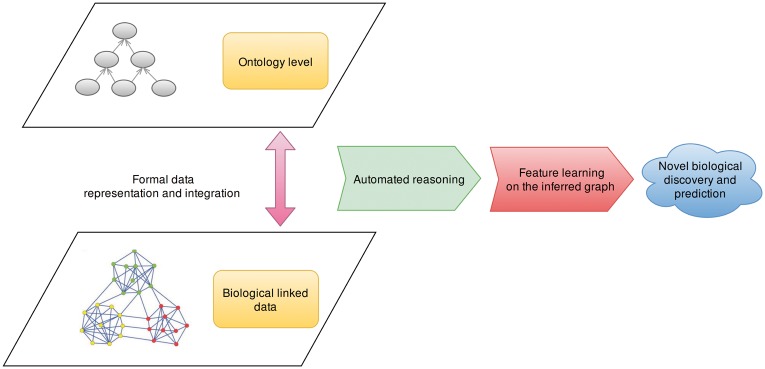
Overview over the main steps in our workflow. We first build biological knowledge graphs by integrating Linked Data, biomedical ontologies and ontology-based annotations in a single, two-layered graph, then deductively close the graph using automated reasoning and apply feature learning on the inferred graph to take into account both explicitly represented data and inferred information. The two layers of the knowledge graph arise from the different semantics of linked biological data (represented in the graph-based language RDF) and the ontologies (represented in the model-theoretic language OWL); we formally connect the entities in the data layer through the rdf:type relation to ontology classes

We automated these steps (ontology-based classification, repeated random walk, generation of embeddings) in an algorithm that combines the steps relying on symbolic inference and the learning of embeddings using a neural network. The input of the algorithm is a knowledge graph and the parameters needed for the algorithm such as the length and number of walks and size of the resulting embeddings, the output is an embedding (of a specified size) for each node in the knowledge graph. Figure 1 illustrates our basic workflow.

### 3.2 Edge prediction

The resulting embeddings can be used in standard machine learning classifiers. We demonstrate these uses in two settings. First, we remove edges from the knowledge graph, regenerate the embeddings using the reduced graph, and train a logistic regression classifier to predict whether or not an edge exists between two nodes, given the embeddings for two nodes as input. This kind of application is intended to demonstrate how associations between two potentially different types of entities, such as a gene and disease, can be identified. We perform these experiments in 5-fold cross-validation setting for every object property in our graph except for edges that exist only between ontology classes. Table 1 summarizes the results. We performed the same experiment using a one-class support vector machine and include results as Supplementary Material.

**Table 1.** Performance results for edge prediction in a biological knowledge graph

Object property	Source type	Target type	Without reasoning		With reasoning	
			F-measure	AUC	F-measure	AUC
has target	Drug	Gene/Protein	0.94	0.97	0.94	0.98
has disease annotation	Gene/Protein	Disease	0.89	0.95	0.89	0.95
has side-effect*	Drug	Phenotype	0.86	0.93	0.87	0.94
has interaction	Gene/Protein	Gene/Protein	0.82	0.88	0.82	0.88
has function*	Gene/Protein	Function	0.85	0.95	0.83	0.91
has gene phenotype*	Gene/Protein	Phenotype	0.84	0.91	0.82	0.90
has indication	Drug	Disease	0.72	0.79	0.76	0.83
has disease phenotype*	Disease	Phenotype	0.72	0.78	0.70	0.77

Object properties marked with an asterisk are between instances and instances of ontology classes.

We find that the performance of the prediction differs significantly by object property, but some object properties can be predicted with high F-measure. Furthermore, using the knowledge graph with reasoning improves the performance slightly when predicting edges between instances and mostly results in decreased performance when aiming to predict edges between instances and instance of an ontology class. We achieve overall highest performance on predicting *has target* edges with an F-measure of 0.94 and ROCAUC of 0.98, and lowest overall performance on associations between diseases and their phenotypes (*has disease phenotype*, ROCAUC 0.77). While our aim here is not to propose a novel method of predicting drug targets, protein functions or phenotypes, our performance is similar to state of the art approaches for related tasks (Wang *et al.*, 2013, 2014). Some of the edges, such as has function or has phenotype, have to be predicted in a hierarchical output space (i.e. an ontology such as the Gene Ontology (Ashburner *et al.*, 2000) and the Human Phenotype Ontology (Köhler *et al.*, 2014)) and need to satisfy additional consistency constraints (due to formal dependencies between the labels), which may overall result in lower performance when applied to these tasks (Radivojac *et al.*, 2013; Sokolov *et al.*, 2013).

### 3.3 Drug repurposing on biological knowledge graphs

As second use case, we also test how well the node embeddings can be used to predict novel relations, i.e. relations that are not explicitly represented in the knowledge graph. Such an evaluation can provide information about how well the embeddings our algorithm generates can be reused in novel applications or as part of larger predictive systems for hypothesis generation (Gottlieb *et al.*, 2011).

We aim to test how much information about shared mode of action is encoded in the embeddings of drug nodes generated by our method, and how the performance of our approach compares to related efforts. Using side-effect similarity alone, it is possible to identify pairs of drugs that share protein targets and indications (Campillos *et al.*, 2008; Tatonetti *et al.*, 2012), thereby demonstrating that side effects provide some information about drugs’ modes of action (Campillos *et al.*, 2008). We train a logistic regression classifier to predict whether a pair of drugs (represented by the embeddings we generate) share an indication or target. To make our input data comparable to studies that compare only drugs’ side effects, and to avoid bias introduced by encoding targets and indications in the knowledge graph, we remove all *has indication* and *has target* edges from our graph and further retain only drugs contained in the SIDER database (Kuhn *et al.*, 2010). We then train a logistic regression classifier to determine whether a pair of drugs shares an indication or a target using 80% of the drug pairs as training and keeping 20% as testing.

**Fig. 2:**
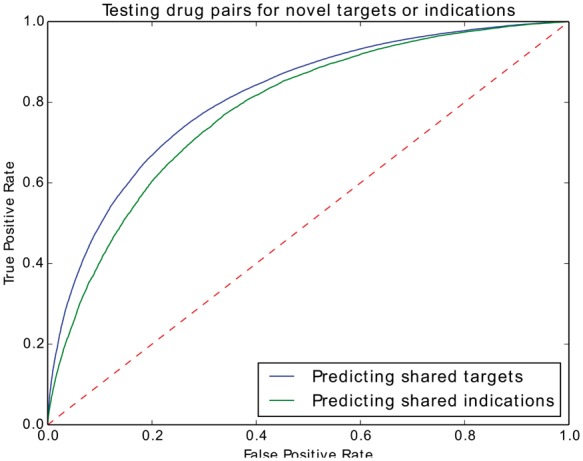
ROCAUC test scores of SIDER drug pairs the for predicting novel indications or targets or both

Figure 2 shows the resulting performance. We can achieve 0.79 ROCAUC for drugs that share targets, and 0.77 ROCAUC for drugs that share indications. In comparison, ranking drug pairs by their side effect similarity alone can achieve a ROCAUC of up to 0.75 for drugs sharing targets and 0.83 for drugs sharing indications (Tatonetti *et al.*, 2012). Our results demonstrate that our method generates embeddings that encode for the explicit information in a knowledge graph, is capable of utilizing this for prediction and achieve comparable results to other approaches. Moreover, after removing *has target* and *has indication* edges, drugs are not directly linked to protein-protein interactions, protein functions or disease phenotypes. Nevertheless, the embeddings generated for drugs based on the corpus generated by random walks can encode some of this information, for example by linking both genes and drugs to similar phenotypes (and thereby providing information about potential drug targets), linking diseases and drugs to similar phenotypes (and thereby providing information about potential indications), as well as more complex interactions.

Instead of using a classifier, similarity between the embeddings can also be exploited to identify biological relations. Using the full knowledge graph, we further tested whether drug-drug similarity can be used to identify drugs that fall in the same indication group. We use cosine similarity to determine how similar two drugs are and evaluate whether drugs that share the same top-level Anatomical Therapeutic Chemical Classification System (ATC) code are more similar than drugs that do not share codes. We find drugs in the same ATC top-level category are significantly ($P<3\cdot 10^{-4}$, Mann-Whitney U test) more similar than drugs that do not fall in the same ATC top-level category.

## 4 Discussion

We present an approach for feature learning on biological knowledge graphs, and demonstrate that these features are predictive of relations between biological entities. Our approach has several advantages over traditional machine learning approaches based on hand-crafted features. First, we reuse existing Linked Data representations of biological databases as well as the OWL ontologies that were developed to characterize their content, and our approach is therefore widely applicable to any kind of biological data represented through RDF and OWL. In the past decades, there have been significant resources committed to the development of linked datasets in biology and biomedicine as well as the development of high-quality ontologies (Jupp *et al.*, 2014; Katayama *et al.*, 2014; Smith *et al.*, 2007), and our work can be applied to these resources and enable or improve data analytics. Our approach also utilizes structured data as well as the ontologies used to capture background knowledge, and through the application of automated reasoning it will therefore not only encode associations between biological entities represented in databases but also their ontology-based classifications, even when these associations are not explicitly stated but inferred. Furthermore, our approach encodes information about network connectivity and communities within a node’s neighborhood in a knowledge graph. The features learned from this information may be used to build prediction models where such information is important, such as gene–disease associations based on the structure of the interactome (Köhler *et al.*, 2008).

We do not demonstrate that we significantly outperform the state of the art in predicting certain biological relations. Our approach has several limitation that affect its performance when used on its own for predicting biological relations. First, machine learning models built using manually crafted features will be able to utilize more specific features that are directly relevant for predicting a particular type of relation. They will also be able to utilize these features better, for example by combining or transforming them so that they can be utilized better to solve a particular problem. Our approach, on the other hand, is not specific to a particular application; we use the same knowledge graph and the same feature learning method for predicting multiple different types of biological relations. Second, our approach only uses qualitative information, while prediction of certain association will often use both qualitative and quantitative information. For example, to predict drug targets, differential gene expression profiles can provide a significant amount of information (Lamb *et al.*, 2006) but we currently cannot incorporate such information in our approach. Third, our approach is approximate in the sense that the neighborhood of a node in the knowledge graph is sampled through a random walk, and in particular for nodes with a high degree of connectivity, information is lost as not all outgoing or incoming edges will be included in a random walk. Despite these limitations, the embeddings that our approach generates can be added as additional features to existing machine learning methods without spending significant effort to manually extract and represent features. The low dimensionality of the embeddings for each node makes our approach particularly suitable for such a combination.

Despite the large success of machine learning methods in the past years (Lecun *et al.*, 2015), they have not yet widely been applied to symbolically represented biological knowledge. Symbolic representations in biology, based on Linked Data and ontologies, are relying on formal languages such as OWL and RDF, and utilize symbolic inference. The kind of inferences performed on this knowledge is either formally specified in the knowledge representation language (Baader *et al.*, 2003) or produced by hand-crafted inference rules that are applicable within a particular database, application, or query (Callahan *et al.*, 2013; The UniProt Consortium, 2015). Here, we use knowledge graphs built using the semantics of OWL and data is represented as instances of OWL classes, but our approach of building knowledge graphs can be replaced with, or amended by, the use of explicit inference rules. In this case, instead of applying an OWL reasoner to infer edges with respect to the OWL semantics, rules can be used to infer edges and deductively close the knowledge graph with respect to a set of inference rules.

A key difference between the knowledge graphs we use in our approach and knowledge graphs widely used in biological databases is the strong focus on representing biological entities and their relations in contrast to representing the (non-biological) meta-data about these entities and their associations, such as provenance (Belhajjame *et al.*, 2012) and authorship. Only few knowledge graphs have been developed that employ such a clear distinction, notably the KaBOB knowledge graph (Livingston *et al.*, 2015). While inclusion of such metadata in knowledge graphs is required for retrieval and to ensure data quality (Wilkinson *et al.*, 2016), our method relies on the use of data models that make it possible to separate the biological content of a knowledge graph from the metadata.

We demonstrate that knowledge graphs based on Semantic Web standards and technologies can not only be used to store and query biological information, but also have the capability to be used for data analysis. The key advantage of choosing knowledge graphs as representation formats for analytical services over other representations is the inherent focus on representing heterogeneous information in contrast to single types of relations, the possibility to continuously add information, the use of inference rules, and the use of World Wide Web standards. Our method allows all these advantages to be utilized and incorporated in predictive models, and may encourage database curators and biologists to increasingly rely on knowledge graphs to represent the biological phenomena of their interest.

## Supplementary Material

Supplementary Data
