# Supplementary material for: Neuro-symbolic representation learning on biological knowledge graphs
Source: Bioinformatics. 2017 Apr 25;33(17):2723–30. doi: 10.1093/bioinformatics/btx275 (PMC5860058; doi:10.1093/bioinformatics/btx275)
Supplement: Supplementary Data [file btx275_supplements.pdf]

Supplementary materials: Neuro-symbolic representation learning on biological knowledge graphs

1 Prediction using one-class SVM

We used absent edges as negatives to train a logistic regression classifier for edge prediction. Due to incomplete information about these biological relations, not all the negatives may be true negatives. We trained a one-class support vector machine (SVM) using only positive instances in a 5-fold cross validation setting for every object property. We optimized the parameters of the one-class SVM through a grid search using the `has indication` object property and included kernel functions (using the linear, polynomial, radial basis function and sigmoid kernels), polynomial function degrees, and gamma values in the search. We found the polynomial function with degree 3 and a gamma value of 0.1 to perform best and used these parameters for all object properties.

**Object property**	**F-measure**
has target	0.877
has disease annotation	0.822
has side-effect	0.830
has interaction	0.737
has function	0.708
has gene phenotype	0.768
has indication	0.722
has disease phenotype	0.713

Table 1: Prediction results using a one-class SVM instead of logistic regression.

2 Degree distribution by object property

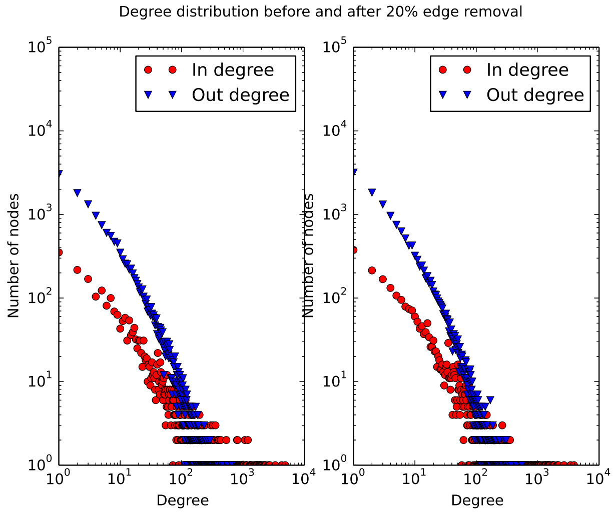

Figure 1: Node degree distribution for `has disease annotation` edges, before and after removing 20% of edges.

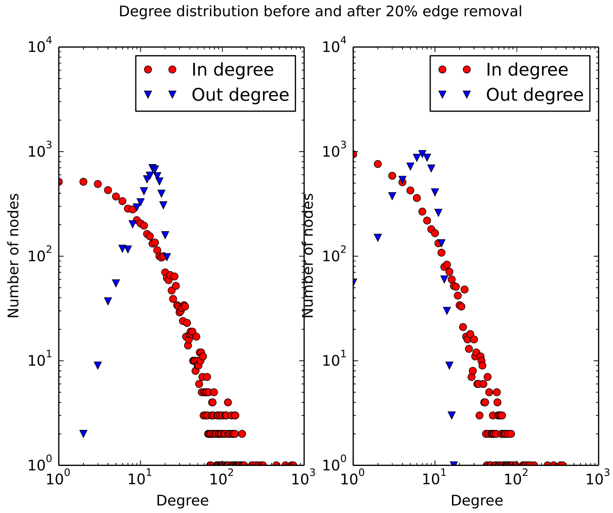

Figure 2: Node degree distribution for `has disease phenotype` edges, before and after removing 20% of edges.

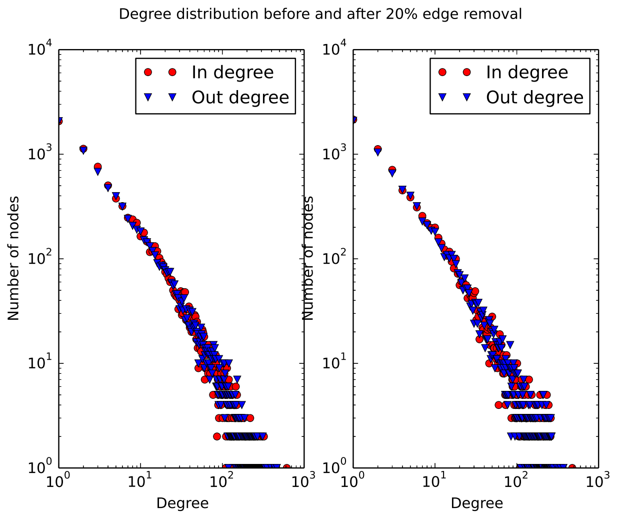

Figure 3: Node degree distribution for `has interaction` edges, before and after removing 20% of edges.

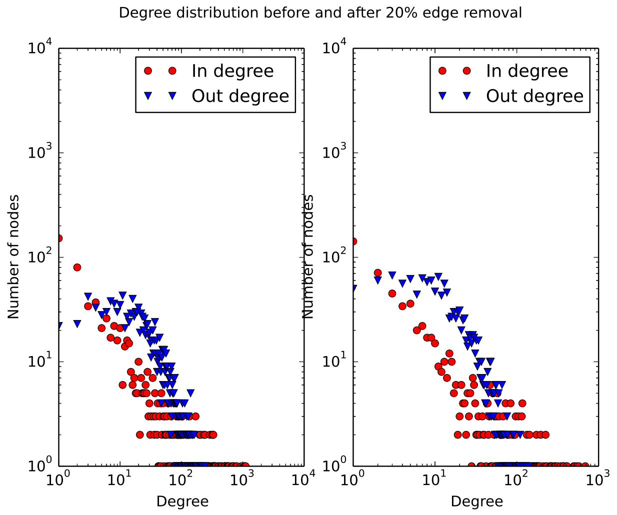

Figure 4: Node degree distribution for `has side effect` edges, before and after removing 20% of edges.

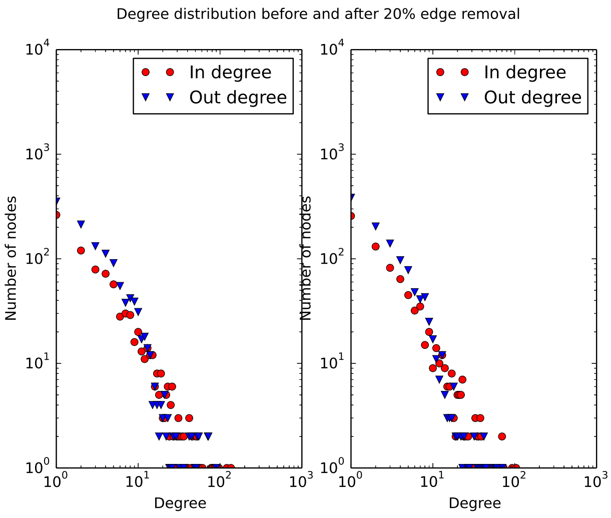

Figure 5: Node degree distribution for `has indication` edges, before and after removing 20% of edges.

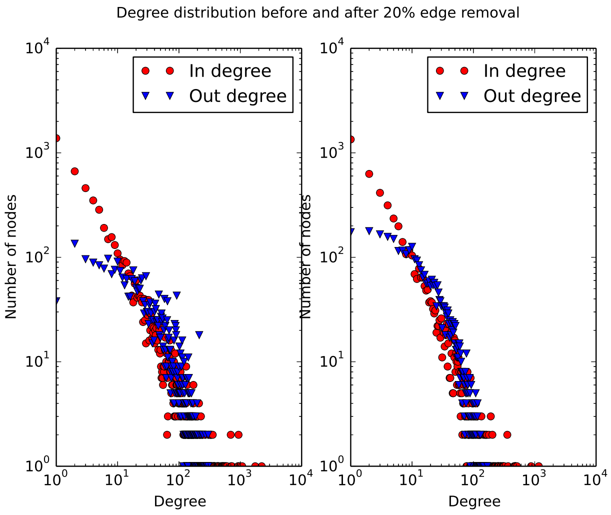

Figure 6: Node degree distribution for `has gene phenotype` edges, before and after removing 20% of edges.

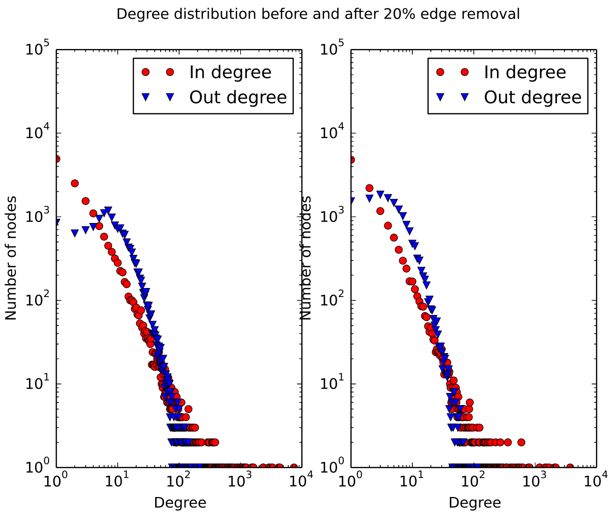

Figure 7: Node degree distribution for `has function` edges, before and after removing 20% of edges.

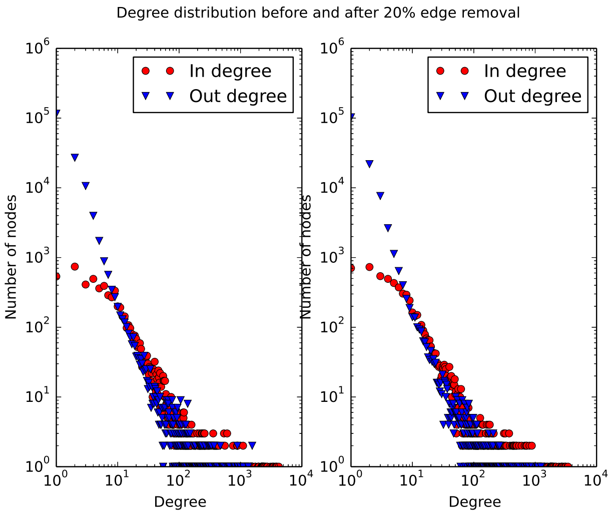

Figure 8: Node degree distribution for `has target` edges, before and after removing 20% of edges.
